# Quality improvement in mental health services

**DOI:** 10.1192/bjb.2019.65

**Published:** 2020-02

**Authors:** Billy Boland

**Affiliations:** Hertfordshire Partnership University NHS Foundation Trust, St Albans, UK

**Keywords:** Quality improvement, quality, mental health services

## Abstract

Quality improvement (QI) approaches are becoming increasingly important in the delivery of mental healthcare internationally. They were originally developed in the manufacturing industry, but the principle of having a systematic approach to improvement has spread to many other industries, not least to healthcare. Quality improvement approaches in healthcare were pioneered in the USA at organisations such as Virginia Mason and the Institute for Healthcare Improvement. In recent years, they have become firmly established in mental health services in the UK's National Health Service (NHS). There are a number of different approaches to quality improvement, but two leading models have taken root: ‘lean thinking’ (also known as ‘lean methodology’ or simply ‘lean’), which arose out of Virginia Mason, and the ‘Model for Improvement’, which came out of the Institute of Healthcare Improvement. This article describes these two quality improvement approaches, critiques their philosophy and explores how they can apply in the provision of mental healthcare, particularly with reference to the use of data, evidence and metrics.

## Lean thinking

Getting to the essence of ‘lean’ is made challenging by the ambiguous language and definitions in the field. Hallam captures this well in observing that the term ‘lean’ has been used interchangeably to describe four different dimensions, specifically ‘the operating philosophy, the tools, the activities, and the state of the manufacturer’ (cited by Stone^1^). In his review on the science of lean, Kyle Stone defines the lean thinking paradigm as the ‘operational philosophy’ of the organisation’ which ‘differentiates between waste and value’.^1^

*Muda* (Japanese for waste) is defined by Womack & Jones as ‘any human activity which absorbs resources but creates no value’^2^ (p. 15). The ‘powerful antidote’ to tackling waste, they argue, is lean thinking. Five ‘lean principles’ are proposed. Data, evidence and metrics, and their application are not specifically addressed within the principles, but their use in realising the principles is clearly important. Definitions of the principles exist, but there are challenges in engaging with them and applying them to healthcare. The next section considers these challenges in relation to the key concept of each principle.

## Challenges engaging with the lean principles

### Specifying value

Womack & Jones argue that value can only be specified by the ‘ultimate customer’^2^ (p. 16), but fall short of clarifying who that is. They make a compelling argument that those who design and deliver the business can never truly know the value that customers seek and must work hard to understand the customer's wants and needs in building their product. How one defines or identifies the ‘ultimate customer’ is not made clear. Instead, readers are encouraged to maintain a dialogue with a broad customer base and continually evolve their product. They offer a number of lenses through which one can see value, such as ‘challenging traditional definitions of value’ (p. 31), defining value in terms of the ‘whole product’ (p. 32) and tackling the ‘target cost’ (p. 35), yet crucially they do not define what value actually is.

Specifying value is key to making best use of data, evidence and metrics, as all efforts should be directed towards improvement aimed at achieving the target ideal value. Having a robust definition of value is necessary in order to demonstrate, using data and evidence, whether or not value has been achieved. Progress towards achieving ideal value can be measured using metrics.

### The value stream

Liker & Ross define the value stream as ‘core customer-focussed business processes’^3^ (p. 241). Womack & Jones offer a more granular explanation:

‘The Value Stream is the set of all the specific actions required to bring a specific product (whether a good, a service, or, increasingly, a combination of the two) through the three critical management tasks of any business: the problem-solving task running from concept through detailed design and engineering to production launch, the information management task running from order-taking through detailed scheduling to delivery, and the physical transformation task proceeding from raw materials to a finished product in the hands of the customer.’^2^ (p. 19)

Both definitions are inadequate as they fail to get to the heart of the matter and they leave important questions unanswered. The Liker & Ross definition is narrow and wholly business oriented. Why should the value stream only regard ‘core’ business processes, and how are these defined? The mass production examples given in both texts do not fully translate to a complex multiservice industry such as healthcare. Which business processes in healthcare are ‘core’? Does that mean ‘non-core’ services do not deliver value?

In contrast, the Womack & Jones definition is too verbose and meaning is obscured as a result. It is also internally incoherent, as although it begins by explaining that a product can be goods or services, later parts of the definition are articulated only in terms of goods, for example ‘the physical transformation task proceeding from raw materials to a finished product’^2^ (p. 19).

In establishing the value stream, obtaining information (data and evidence) from those involved in delivering the work is crucial to truly understanding what is happening. Evidence from customers (such as patients and carers) is important in understanding what value is actually delivered at relevant points through the stream. Metrics can be used to indicate whether value has been delivered, provided that the value has been clearly specified. For example, in mental health services patient-reported outcome measures (PROMS) and patient-reported experience measures (PREMS) can give some insight into patients' experience of services and service impact.^4^

### Flow

Frustratingly, a ‘lean’ definition of flow is hard to come by, and most authors appear to have resorted to explaining what it is not. Womack & Jones explain that it is not batch processing or doing tasks in batches that inherently cause waiting and queuing^2^ (p. 50). In their analysis of lean in an emergency department in an NHS hospital in Nottingham, Timmons and colleagues talk of the lean principle in the healthcare setting in terms of ‘ensuring that there is a continuous flow throughout the process. Standardising processes around best practice allows smooth running, which frees up time for creativity and innovation’.^5^

Flow can be quantified with respect to value and so can be amenable to the generation of metrics. Indirect measures of waste (*muda*) are useful data and evidence proxy indicators for flow. For example, measures of defects, overproduction or waiting can all give useful insights into the performance of flow^6^ (pp. 82–84).

### Pull

Pull also seems to be defined by what it is not. For example, Womack & Jones offer: ‘you can let the customer pull the product from you as needed rather than pushing products, often unwanted, onto the customer’^2^ (p. 24) and ‘pull in simplest terms means that no one upstream should produce a good or service until the customer downstream asks for it’^2^ (p. 67). Although, on the face of it, pull seems a wholly transactional concept, Liker & Ross emphasise ‘even a well-designed pull system does not automatically solve all our problems and is dependent on human judgement and discipline’^3^ (p. 177). Once more in these definitions we see a focus on goods rather than services, and so aspects of the human nature of pull are obscured. For example, in their new study of lean in a Finnish healthcare service, Hihnala and colleagues state that, although work and workflow can be ordered to create pull, ‘It emerged […] that a common set of values that respects human dignity came [sic] more important’.^7^

Pull too can be amenable to metrics to help maximise value. Measures of demand can be a useful source of data, which can be used to evidence how to manage the flow. Qualitative sources of evidence can include customers' (patients' and carers') accounts of their experience and motivations to make use of the service^6^ (p. 96).

### Perfection

This is yet another concept with a shadow side. Womack & Jones once again avoid defining it, but talk of it as being the continuous application of the previous four principle concepts to strive to continue achieving better products^2^ (p. 25). Perfection is never achieved, but there is always room for improvement. Actually, what they are truly referring to is the continual pursuit of identifying and eliminating waste (*muda*).

Metrics are important here as perfection needs to be defined. Perceptions of perfection can change over time, so descriptions need to be clear and revised regularly. Perfection may be best approached incrementally (monitored through metrics) and is never reached. Evidence demonstrating approaching perfection can be quantitative (e.g. in consistency of data) or qualitative (e.g. in having a culture free of fear that promotes quality improvement).

## Lean in practice: a case example

Tees, Esk and Wear Valleys NHS Foundation Trust (TEWV) were supported by Virginia Mason in the USA in adopting lean.^8^ Virginia Mason's own lean processes were adapted from the Toyota Production System, with a strong focus on eliminating waste (*muda*) and the use of metrics to measure improvement.^9^ TEWV have made a number of high-impact changes to the way their processes operate that have had benefits (brought value) to patients. One of these, the purposeful in-patient admission (PIPA) model, which was trialled on two adult wards, saw their original bed occupancy of 106% reduce by 22%, a 57% reduction in length of stay, a 72% reduction in reports of violence and aggression, and a 100% reduction in complaints (p. 62).^10^

Lean techniques they used to achieve these changes included:

- a move from a weekly ward round to daily multidisciplinary team meetings – to remove ‘batching’ of decision-making (to improve *flow* of the *value stream* of clinical decisions and interventions)
- visual control boards on the wards – to map the patient journey (*flow*)
- removal of waste (*muda*) from the office and patient literature – ensuring that only necessary literature were on the ward
- creating standard processes for each step of the patient journey and changing the layout of the ward environment (to improve *flow*).

The Health Foundation is now exploring how other organisations might benefit from lean and is conducting research into the effect a partnership with Virginia Mason would have on five other NHS organisations.^11^

## Lean thinking – evidence of impact

Although there are countless books and journal articles on the subject, Stone^1^ identifies three ‘voids’ that lean research is yet to adequately tackle:

- the relationship between planned organisational change and human resource development interventions
- the relationship between planned organisational change theories and lean theories
- the ‘human’ factor – by this he means how best to engage staff in lean.

In contrast, in *The Toyota Way*, Liker defines the tenth of the 14 management principles of Toyota as ‘develop exceptional people and teams who follow your company's philosophy’^12^ (p. 184) and explains that at the company the Toyota Production System was originally called the ‘Respect for Humanity System’^12^ (p. 186). Nevertheless, other than his own observations, and a critique of theories of behaviour, Liker offers no empirical evidence for human resources and the human factor.

## The Model for Improvement

Don Berwick, the improvement champion who has led the Institute of Healthcare Improvement (IHI) in the USA and who was called on by the then Prime Minister David Cameron to help make the NHS safe for the future^13^, writes an impassioned introduction for *The Improvement Guide*, a key text that describes the Model for Improvement.^14^ Notably though, the authors of the guide, who Berwick says he is still learning from, are largely statisticians by training. This is important, as the focus in lean is on waste and flow, whereas data, and the use of data, are at the heart of the Model for Improvement.

Berwick calls the model the ‘most useful single framework’ he has encountered^14^ (p. xiii). Founded on the work of Deming, also a statistician, the ‘System of Profound Knowledge’ is fundamental to the Model of Improvement. The system asserts that, through developing appreciation for a system, understanding variation (data, evidence and metrics are clearly important here), building knowledge and getting to grips with the human side of change, one is better equipped to make improvements^14^ (p. 76). Applying the Model for Improvement to an improvement challenge helps the leader organise an approach by guiding them through the following key questions.

- ‘*What are we trying to accomplish*?’ is about being specific and defining the problem. This makes it measurable and potentially subject to metrics. The question is also used as a call to arms, by clearly describing what is expected, and to create ownership, through encouraging others to contribute to creating this definition or ‘charter’^14^ (p. 89). There are some darker sides to how this statement is used to motivate change, of the stick rather than carrot variety. For example, Langley and colleagues^14^ suggest choosing goals that are clearly unachievable using current practices, being explicit that previous tools will be removed whether change happens or not, or simply stating that the current service will no longer be provided if change does not happen. Thus, although values of addressing the ‘human side’ of change are espoused, the authors are not shy of using traditional ‘machine metaphor’ classic top-down management theory^15^ (p. 18) if they think it will help towards achieving the goal. This seems to be in contrast to the approach Berwick advocates in his work on patient safety for the NHS, where he says, ‘Fear is toxic to both safety and improvement’.^13^
- ‘*How will we know that a change is an improvement*?’ is sometimes abbreviated to ‘measures’. This is all about how to demonstrate the impact of change using data, evidence and metrics. Three different types of measures are encouraged: outcome measures that observe the outcome in question, process measures that monitor whether activity to achieve the outcomes is performed, and balancing measures that look at whether there are any unintended consequences of change^14^ (p. 96). One could argue that the model encourages only superficial engagement with the ‘is the change an improvement?’ part of the question by focusing on measures. For example, in a healthcare system where increasing discharge is the aim, is there enough challenging of the assumption that discharge is the right thing?
- ‘*What change can we make that will result in improvement?*’ is often abbreviated to ‘changes’ and is about identifying initiatives that could bring about change^14^ (p. 93). Methods for developing change are promoted, including ‘logical thinking about the current system, benchmarking or learning from others, using technology, creative thinking and using change concepts’^14^ (p. 120). ‘Changes’ can be opportunities to put evidence into practice, and could be an application of evidence-based medicine, such as the implementation of National Institute for Health and Care Excellence (NICE) guidance.^16^ Dozens of change methods are proposed that can be used in the Model for Improvement and many of these, such as ‘use pull systems’, ‘eliminate things that are not used’ and ‘match the amount to the need’, have clear roots in lean thinking^14^ (p. 358). Langley and colleagues acknowledge the overlap with other improvement approaches: ‘Several of the concepts are included in other approaches to improvement, such as Total Quality Management, Reliability, Safety, Six-Sigma, and Lean’^14^ (p. 358).

With the three Model for Improvement questions answered, improvers are ready to make use of the ‘plan–do–study–act’ (PDSA) cycle. PDSA can be used to ‘turn ideas into action and action into learning’^14^ (p. 97). There are four distinct phases to PDSA:

1. the intervention or test should be planned
2. the plan should be executed and data recorded
3. data are analysed
4. reasonable action is taken on the findings (essentially action is based on evidence).

The cycles of PDSA can be used to ‘build knowledge’ both of the improvement challenge faced and potential solutions.^17^ PDSA cycles are recommended by NICE to bring about improvements through implementing NICE recommendations.^16^ A model akin to ‘plan–do–study–act’, called ‘plan–do–check–act’, has been used in lean in, for example, work on patient safety.^18^

## The Model for Improvement in practice: case examples

East London NHS Foundation Trust (ELFT) adopted the Model for Improvement, supported by the IHI. The trust's work to reduce violence on in-patient wards saw a 40% reduction in violence across six wards and reduced costs related to violence by £181 296 (data are for 2015–2016).^19^ The Care Quality Commission (CQC), the UK's healthcare regulator, has rated the organisation as ‘outstanding’ and commented:

‘ELFT has invested over the previous two years in a wide scale quality improvement programme. This has been embraced by staff. The methodology has successfully encouraged innovation and improvement which CQC inspectors were able to see throughout the inspection. There was a genuine passion to ensure that the services provided are the best possible.’^20^

The teams used the Model for Improvement questions to define and drive their work. They agreed what they wanted to *accomplish* (to reduce physical violence by 30%) and the *measures* to determine whether a change was an improvement (the main outcome measure used was ‘rate of incidents of physical violence per 1000 occupied bed-days’). In generating ideas for change, the team worked with staff and patients, and then used PDSA cycles with wards from across the trust to test the favoured change strategies. Staff came together at 6-weekly intervals to learn from each other and review data to consider whether improvement was happening.^21^

Hertfordshire Partnership University NHS Foundation Trust has also employed the Model for Improvement, launching it in 2015. Subsequently, the 2016 national NHS staff survey reported that the percentage of staff in the trust who said they were able to contribute to improvements had increased to 76%, from 70% the previous year. The Picker Institute, which analyses the data for the NHS, reported this as a statistically ‘significant improvement’ and above the national average for mental healthcare.^22^ The trust was also rated ‘outstanding’ by the CQC in May 2019. It said:

‘The delivery of innovative and evidence based high quality care was central to all aspects of the running of the service. There was a true sense of desire to drive service improvement for the benefit of patients, carers, and the wider system, evident throughout the inspection. Staff included patients in service improvement and used their feedback to change practice.’^23^

## Model for Improvement – evidence of impact

Evidence for PDSA in a UK healthcare context is varied. A systematic review of quality improvement methodologies carried out by NHS Scotland found that the evidence for PDSA in the NHS was ‘mixed’. It found that, in large projects taken forward by the NHS Modernisation Agency, work often did not proceed beyond ‘plan–do’. Other initiatives, such as work on the 4-hour emergency department (A&E) waiting target, could lead to problems elsewhere in the system (such as the patient waiting in a medical assessment unit instead). Success can be context dependent, with ‘striking differences’ between organisations using PDSA in quality improvement collaborations.^24^ Furthermore a multisite cluster-randomised study comparing the effectiveness of different change initiatives within healthcare services in the UK found no difference between standard dissemination of guidance versus standard dissemination plus PDSA in the implementation of the guidance.^25^

## Conclusion – the essence of theories of improvement science

Considering these two approaches to quality improvement, a number of factors emerge as the essence of these theories:

- They aim to be pragmatic, seeking to clearly describe the problem and bring about real-world change.
- Although they draw on scientific theory, they are about implementing and applying scientific method to bring about improvement.
- The sensible use of data, evidence and metrics is essential in order to demonstrate change.
- They are about both processes and people. Automation can enhance productivity, but it is people that bring about change.
- They are sensitive to context. The evidence base demonstrates that improvement methods can succeed and fail, but identifying win factors can be a challenge. Leadership, engagement and culture are all extremely important.
- They are not discrete. The various improvement methods have considerable degrees of overlap and borrow from each other, for example lean can employ a ‘plan–do–check–act’ cycle, the Model for Improvement can use lean concepts.

The Health Foundation has summarised a similar list of ‘underlying principles’, which include understanding the problem, understanding the processes and systems, analysing demand, capacity and flow, choosing tools for change and evaluating change^26^ (p. 11). In September 2018, the CQC published a report into what it had learned about embedding a quality improvement culture within healthcare organisations. It states, ‘We would expect that a hospital trust committed to delivering high-quality care should be embedding a systematic and effective approach to QI’.^27^ Regardless of the provenance of the approaches and their evidence base, in the UK at least, it looks like they are here to stay for the foreseeable future. Clinicians will benefit from understanding quality improvement and could do much to shape how it is received and applied in their context.

## About the author

**Billy Boland** is a Consultant Psychiatrist in community psychiatry and Deputy Medical Director at Hertfordshire Partnership University NHS Foundation Trust, United Kingdom. He is the current Chair of the General Adult Faculty of the Royal College of Psychiatrists.
